# Propositions in Regard to Cancroid

**Published:** 1855-02

**Authors:** Paul Broca


Propositions in Regard to Cancroid. By Paul Broca.—

1. Cancroid is an homœomorphous tumour constituted by the elements of epithelium.

2. Cancroid tumours are distinguished from cancers by two series of characteristics; those, namely, which are determined by the naked eye, and those which are revealed by the microscope. The lesions of these two diseases are different, pathological anatomy consequently compels us to separate them.

3. The local development of cancroid presents many analogies with that of cancer, but we observe differences in the manifestations of the two diseases, differences which consider individually are perhaps insufficient to distinguish them, but which taken together form a group of characteristics as precise as any purely clinical characteristics can possibly be.

4. Cancroid is very often developed under the influence of purely local causes, such as slight but frequently repeated irritations; many surgeons deny the influence of local causes on the development of cancer, and it is certain that if it is ever affected by causes of this sort, it is only in exceptional cases.

5. Cancer is developed under the influence of a general diathesic cause. It is not so with cancroid. It is true, that in very rare cases, two or more cancroids may appear simultaneously or successively in the same individual. Their presence on several distinct portions of the cutaneous system is an evidence of the existence of a sort of diathesis in this system, just such as we observe when successive crops of warts are thrown out on the skin, just such as exists in many other purely local diseases. But no one can confound these local diatheses, limited to a single organic system, with the general diatheses, among which the cause of cancer is included.

6. Hereditary predisposition, which is often the cause of cancer, has not been hitherto found to have any connection with the production of cancroid. Undoubtedly, it is not impossible that two individuals of the same family should both suffer from cancroid tumours; the calculus of probabilities indicates that this should occasionally occur; but these cases are as yet hypothetical, whereas the influence of hereditary predisposition in cancer is based on numerous and precise facts, not to be explained away as simple coincidences.

7. Cancroid only appears on parts covered by epithelium.

Cancer, on the contrary, may commence anywhere that there are vessels.

8. Many organs in which cancer rarely appears, are often the seat of cancroid. Many organs in which cancer is common are never the seat of cancroid.

9. True cancer of the skin is very uncommon. The majority of the suspicious affections of the skin formerly designated cancer of the lip, breast, prepuce, &c., are nothing but cancroids.

10. The progress of cancroid is usually much slower than that of cancer. The duration of life when no operation is performed is much longer in cancroid than in cancer; longer in the proportion of 10 to 1 almost.

11. Cancer ordinarily commences by a circumscribed tumour; cancroid usually appears first as a button or pimple with a large base, as a wart, or as a papillary hypertrophy; or else as an excavated, scarcely indurated surface, covered for a long time by successive crusts, before it becomes the seat of a true ulceration.

12. These characteristics are so marked, that clinical observers have always been struck by them, and have thought it proper to bestow a particular name on the tumours which we now call cancroids.

13. Cancerous ulcers give rise to much more frequent and abundant hæmorrhages than are produced by the ulcers of cancroid.

14. Cancer invades the glands more uniformly and more rapidly than cancroid.

15. Cancer and cancroid, when let alone, usually terminate in death; but in the case of cancroid death is the result either of the seat of the tumour, as in cancroid of the larynx, of exhausting suppuration, or of putrid absorption, there is nothing special in it. In cancer, death may arise from these same causes; but sooner or later there is produced besides a specific morbid condition, the cancerous cachexy, or infection.

16. Both cancer and cancroid may relapse after extirpation, but an examination of these relapses points out new differences in these affections.

17. It is demonstrated that relapse is infinitely more common in cancer than in cancroid. This truth has been recognized and proclaimed in every age, long, long before the introduction of the microscope in pathological enquiries.

18. It is certain that a great number of cancroids are cured by the knife; it is doubtful whether true cancer has ever been cured.

19. Cancroids only relapse near the site of their first appearance (par continuation) that is to say within the sphere of local action of the primary tumour. They appear either immediately on the site of the preceding tumour, in its vicinity, or on neighboring glands, and this only exceptionally. Cancer often reappears in the same manner, but it also buds forth very frequently in distant parts of the organism.

20. In about every other case of cancer, we find, at the autopsy, cancerous infiltration in the internal organs; nothing of the sort is observed after death from cancroid. More than fifty examinations have been made in France of bodies of persons dying with cancroid, and in not one have multiple cancroids been detected; the unique exception quoted by Mr. Paget, even admitting it to be incontestable, by its extreme rarity, contrasts in the most striking manner with the extreme frequency of cancerous infection.

21. Cancroid is a tumour, cancer is a disease; the first is the result of a local affection of the cutaneous system, the second is due to a general affection of the economy.

22. The distinction between cancroid and cancer has been suspected, hinted at, and even treated of by numerous surgeons of the past century. Pathological anatomy only confirms and renders incontestable a distinction recognized by pathology proper.—*Mémoires de l'Académie de Médecine.* Tome XVI.

